# Modeling Living Cells Within Microfluidic Systems Using Cellular Automata Models

**DOI:** 10.1038/s41598-019-51494-1

**Published:** 2019-10-17

**Authors:** Julia Ballesteros Hernando, Milagros Ramos Gómez, Andrés Díaz Lantada

**Affiliations:** 1 Laboratorio de Desarrollo de Productos, Departamento de Ingeniería Mecánica, Universidad Politécnica de Madrid, c/José Gutiérrez Abascal 2, 28006 Madrid, Spain; 2 Centro de Tecnología Biomédica, Universidad Politécnica de Madrid, Parque Científico y Tecnológico, M40, Km. 38, 28223 Pozuelo de Alarcón, Madrid Spain

**Keywords:** Computational models, Biomedical engineering

## Abstract

Several computational models, both continuum and discrete, allow for the simulation of collective cell behaviors in connection with challenges linked to disease modeling and understanding. Normally, discrete cell modelling employs quasi-infinite or boundary-less 2D lattices, hence modeling collective cell behaviors in Petri dish-like environments. The advent of lab- and organ-on-a-chip devices proves that the information obtained from 2D cell cultures, upon Petri dishes, differs importantly from the results obtained in more biomimetic micro-fluidic environments, made of interconnected chambers and channels. However, discrete cell modelling within lab- and organ-on-a-chip devices, to our knowledge, is not yet found in the literature, although it may prove useful for designing and optimizing these types of systems. Consequently, in this study we focus on the establishment of a direct connection between the computer-aided designs (CAD) of microfluidic systems, especially labs- and organs-on-chips (and their multi-chamber and multi-channel structures), and the lattices for discrete cell modeling approaches aimed at the simulation of collective cell interactions, whose boundaries are defined directly from the CAD models. We illustrate the proposal using a quite straightforward cellular automata model, apply it to simulating cells with different growth rates, within a selected set of microsystem designs, and validate it by tuning the growth rates with the support of cell culture experiments and by checking the results with a real microfluidic system.

## Introduction

Recent progresses in the design, prototyping and manufacturing of microfluidic systems^1,2^ have enabled new ways to approach the study of disease, with the advent of lab-on-a-chip technologies that integrate several lab operations in single microfluidic networks, and to advance in the comprehension of cell-cell and cell-material interactions, with the engineering of organ-on-a-chip (O-o-C) devices that mimic the physiological response of entire organs and systems by employing multi-channel cell culture chips^3,4^. These models are starting to replace more common cell culture systems, mainly Petri dishes, as the multi-channel structure provides cells with a 2D^1/2^ or 3D environment more similar to the actual *in vivo* configurations.

In spite of the impressive advances achieved in the field of organs-on-chips in the last decade, mainly in connection with prototyping and validating the viability of these organ-on-chip systems as relevant research tools for studying complex pathologies in a sustainable and systematic way, there is still place for performance optimisation. For example, the successful integration of organ-on-a-chip devices into completely functional humans-on-chips is still matter of research, as happens also with the need for systematic engineering design processes oriented to these types of devices, in which extensive use of simulation techniques may help to optimise the design and channel configurations, among other challenges^5,6^. Until now, the application of simulations to improve the design process of these systems, mainly resorting to finite-element modelling (FEM) has proven useful^7,8^, although the simulation of cell growth and interaction within these systems is not so common.

In fact, being the eukaryotic cell an extremely complex micro-cosmos in itself, simulating its behavior and the interactions with companion cells and extra-cellular environment, so as to model their *in vivo* performance and hence advance in our understanding of disease, constitutes a long-pursued objective and a current research challenge in the intersection between engineering, medicine, basic and biological sciences with varied approaches^9,10^. Modelling to collective behavior of cells within *in vitro* culture environments is also a complex issue, usually performed by means of discrete cell models, typically cellular automata and cellular automata-like models (i.e. cellular Potts, Glazier-Graner, agent based, among others)^11,12^. These discrete models have some drawbacks when compared to continuum approaches, including computational cost for larger cell numbers and precise lattices and need for calibration upon macroscopic measurements. However, discrete models can be more easily fine-tuned by means of averaged measurements from controlled experiments, when the model parameters from continuum models are related to difficult-to-measure cell scale phenomena^12^. In this study we focus on modelling collective cell behavior by using discrete cell models, whose origins and applications to modelling cell colonies are detailed below.

Going to the origins of modern discrete modelling, cellular automata were developed on the basis of work by pioneers, such as Stanislaw Ulam and John von Neumann, as a collection of elements or cells defined upon a grid that evolves through time steps following a set of rules applied iteratively. Along the time steps, the state (i.e. colour or value, typically “0” or “1”) of the cells within the grid changes according to the rules and to the previous states of neighbor cells^13^. Since the beginning, these models were conceived as possible simulators for biological systems and well-known examples of application appeared, such as Conway’s game of life^14^, in which the cells upon a two-dimensional grid have two possible states, dead or alive, and in which cells survive, reproduce, die by under- or over-population, depending on the 8 neighboring cells or the previous generation. Apart from the initial game-like demonstrations, further studies led to verifying that extremely complex systems could be modeled by using cellular automata^15^.

More recently, in the specific area of modelling cell behavior, cellular automata have been used for modelling cell adhesion and proliferation;^16^ for modelling migration, proliferation and differentiation^17,18^; or, in connection with lattice-Boltzmann methods, to model multi-scale avascular tumor growth coupled with nutrient diffusion and immune competition^19^. As for other discrete cell models working upon lattices, the cellular Potts model^20^ complements the lattice with an energy function or Hamiltonian that can be defined to control different cell behaviors, including migration, clustering and growth, and to add volume and surface constraints to the model. This approach has led to the implementation of CompuCell3D^21^, one of the most used software worldwide for modelling cells and their collective behavior, which has been employed for modelling cancer growth and invasion^22^, to simulate epithelial-mesenchymal transitions^23^, and also as educational tool for biomedical engineering degrees^24^, to cite just some examples selected among dozens of publications available in the CompuCell3D website (http://www.compucell3d.org).

In any case, all these discrete cell models used for predicting collective behaviors normally operate on infinite or boundary-less 2D lattices, hence modelling cell growth, migration, death and interactions in “Petri dish” like environments, with the same limitations as the use of Petri dishes for understanding *in vivo* performance using an *in vitro* approach. To the best of our knowledge, these cellular automata and cellular automata-like models have not yet been applied to modelling cells within lab- or organ-on-a-chip devices, which could support the systematic design and optimisation of these sorts of innovative biomedical devices and their steady regulatory compliance verification, by means of selected experiments and exhaustive simulations.

Consequently, in this study we focus on the establishment of a direct connection between the computer-aided designs (CAD) of microfluidic systems, especially labs- and organs-on-chips (and their channel structures), and the lattices for discrete cell modelling approaches aimed at the simulation of collective cell interactions, whose boundaries are defined directly from the CAD models. We illustrate the proposal using a quite straightforward cellular automata model, apply it to simulating cells with different growth rates, within a selected set of microsystem designs, and validate it by tuning the growth rates with the support of cell culture experiments and by checking the results with a real, although simple, microfluidic system. The materials and methods employed are detailed further on, before presenting and discussing the key results.

## Materials and Methods

### Creating the grid and boundaries for a cellular automata model from the CAD file

Throughout the study we use NX-8.5 (Siemens PLM Solutions) for computer-aided design purposes –mainly for designing the microfluidic systems and organ-on-a-chip devices– and Matlab (The Mathworks Inc.) for developing the code of the cellular automata model working upon such microsystems and performing the collective cell simulations. As mentioned earlier, a key objective of our model is to directly link the computer-aided designs of organ-on-a-chip devices with the lattices used for the cellular automata model. Accordingly, the channels and chambers of the microsystems should define the allowed cell positions limited by the vertical walls, which prevent cells from escaping the microsystems, as they operate typically closed by a microscope glass cover slip in real applications.

**Figure 1:**
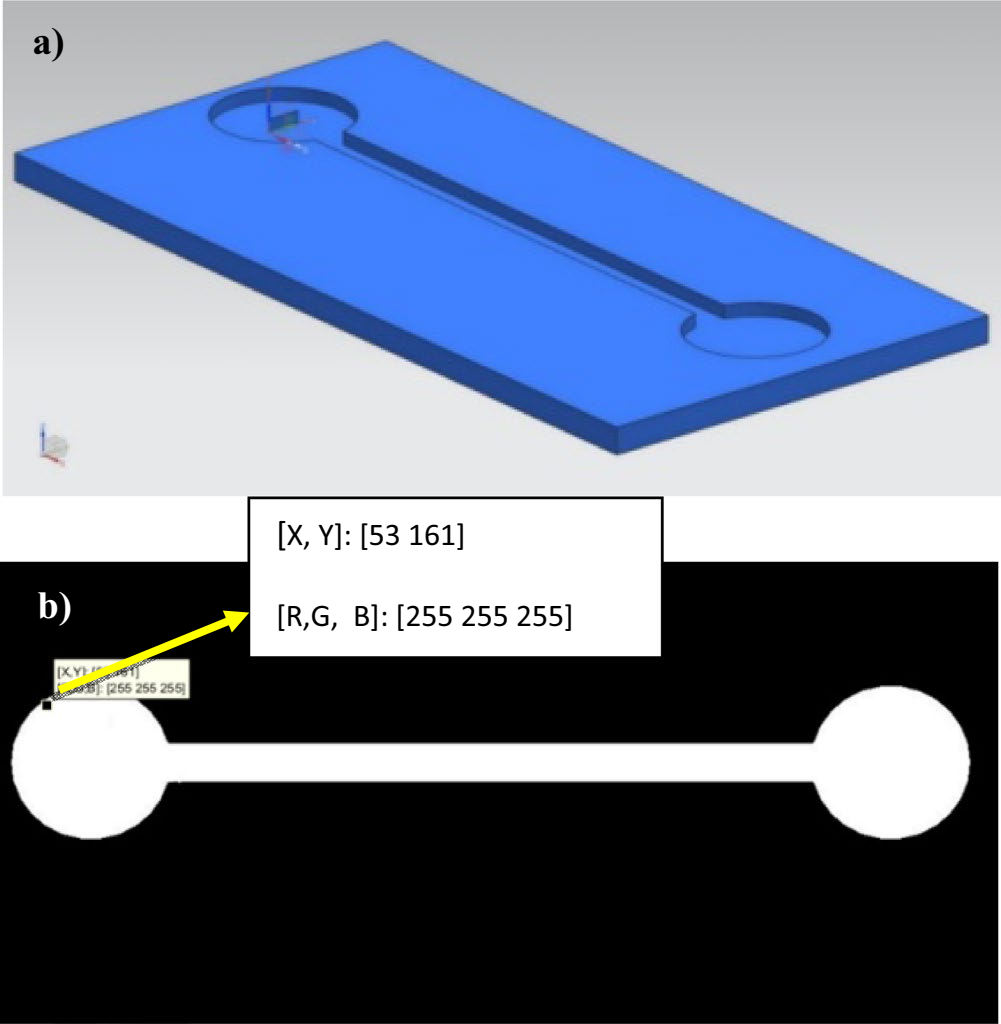
(**a**) Computer-aided design of a simple microfluidic device. (**b**) Boundaries for the lattice-based simulation, in which the white pixels represent allowed regions for the cells (one cell-one pixel), while the black zone is prohibited and not considered for the simulation.

Such a connection can be performed in a quite straightforward fashion: Starting with the 3D CAD file of the microfluidic system, the model is positioned to show a top view, whose surfaces are painted using “paint operation” tools, so as to display the channels in white and the boundaries in black. A final conversion into.jpeg format enables direct import using Matlab as working lattice. The process has direct connections with the generation of digital masks, directly from CAD files, for the manufacture of microfluidic devices by mask-less UV-photolithography^25^. Figure 1 provides a simple example by showing the computer-aided design of a device, made of a single channel connecting an inlet and an outlet, and the obtained boundaries for the lattice-based simulation, in which the white pixels represent allowed regions for the cells (one cell-one pixel), while the black zone is prohibited and not considered for the simulation. As can be seen, each pixel or point of the lattice is defined by its position [X, Y] and by a colour in [R G B] format. The colour helps to distinguish between prohibited lattice points (black), empty lattice points (white) or lattice points with different cell types (i.e. green and blue), with dead cells (red) or cells affected by a drug (pink), to cite some options.

In order to avoid scale problems, the size of the mask should be carefully selected according to the real size of the microsystem and to the actual size of the cells under study, which defines each pixel, in our case corresponding to a square of some 20 × 20 μm^2^. Having a pixel per cell may be in some cases computationally expensive, so an alternative option would be to generate coarser lattices and to work with cell aggregates. Besides, in some cases a fine-tuning of the generated lattice, which derives from the digital mask created from the CAD file, is needed before starting the simulations. The reason is that sometimes the boundaries between the allowed (white) and prohibited (black) zones are not so sharp, due to exporting from CAD to a.jpeg file, and some slightly grey borders appear, which should be transformed to real white [255, 255, 255] (or alternatively to real black [0, 0, 0]) by automated loop inspection of the generated hyper matrix and substitution of all pixels with colour values different from [0, 0, 0] or [255, 255, 255] by [255, 255, 255] (or [0, 0, 0]). Regarding the incorporation of 3D CAD models into Matlab workspace, other Matlab functions may be used to complement the imported.jpeg images that define the boundaries of the cellular automata.

### Modelling cell behaviors with the cellular automata model

**Figure 2:**
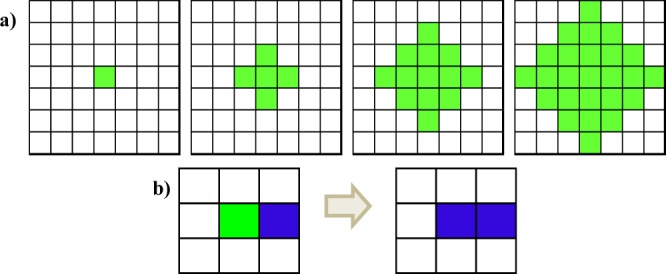
Example of different evolutions along consecutive time steps. (**a**) A single cell reproduces making the adjacent positions become populated. (**b**) A normal cell is transformed into an abnormal state by the presence of an invasive cell.

Summarizing, our cellular automata working on chips follows and enables the following steps and operations: a) establishes allowed zones of the microsystem for the cells being cultured and simulated (those pixels with RGB values equal to: [255, 255, 255]); b) models cell proliferation, as in Fig. 2a, using equation: [eq. 1] ${y}_{n}={y}_{n-1}+(4\times n)$, for the number “y_n_” of cells at time step “n”; c) may incorporate cell death by adding a probability of cell death (Pd) from one time step to another, hence leading to a modified proliferation equation: [eq. 2] ${y}_{n}={y}_{0}+{\sum }_{1}^{n}(4\times n\times (1-{P}_{m}))$ with *y*_0_ = 1; d) models interactions between different cell types, defined with different colors, enabling the simulation of invasive cells (i.e. blue) that convert surrounding healthy cells (i.e. green) in invasive cells, as in Fig. 2b; and e) models the addition of a substance, drug or reactive, which diffuses following a proliferation equation similar to the one already discussed but with another dynamic, as it is defined to interact with a specific type of cell line. Cell migration is not considered in this first model implementation, as the cells we would be testing (see Section 2.4) are of adherent nature, but could be of direct implementation to achieve a more universal simulator, which could be also complemented with energetic functions similar to those used in the cellular Potts model and related ones. For the purpose of connecting CAD models with cellular automata with defined boundaries for simulating collective cell behaviors within organ-on-chip devices this preliminary approximation may prove sufficient. A simulation starts by defining the initial positions of the microsystem where the cells are placed, either by modifying pixel color of the desired positions by writing within the lattice matrix or by clicking in the *ad hoc* developed interface to position cells of different types and eventual substances, reactives or drugs. Each step goes on by evaluating the allowed positions in loops along x and y directions, by leaving white positions without neighboring cells white, by evaluating the probability of death of each cell (and eventually transforming those dead into red), by making healthy cells proliferate according to scheme 2a and by taking account cell-cell interactions, according to scheme 2b.

### Set of computer-aided designs and prototypes of microsystems for testing the model

**Figure 3:**
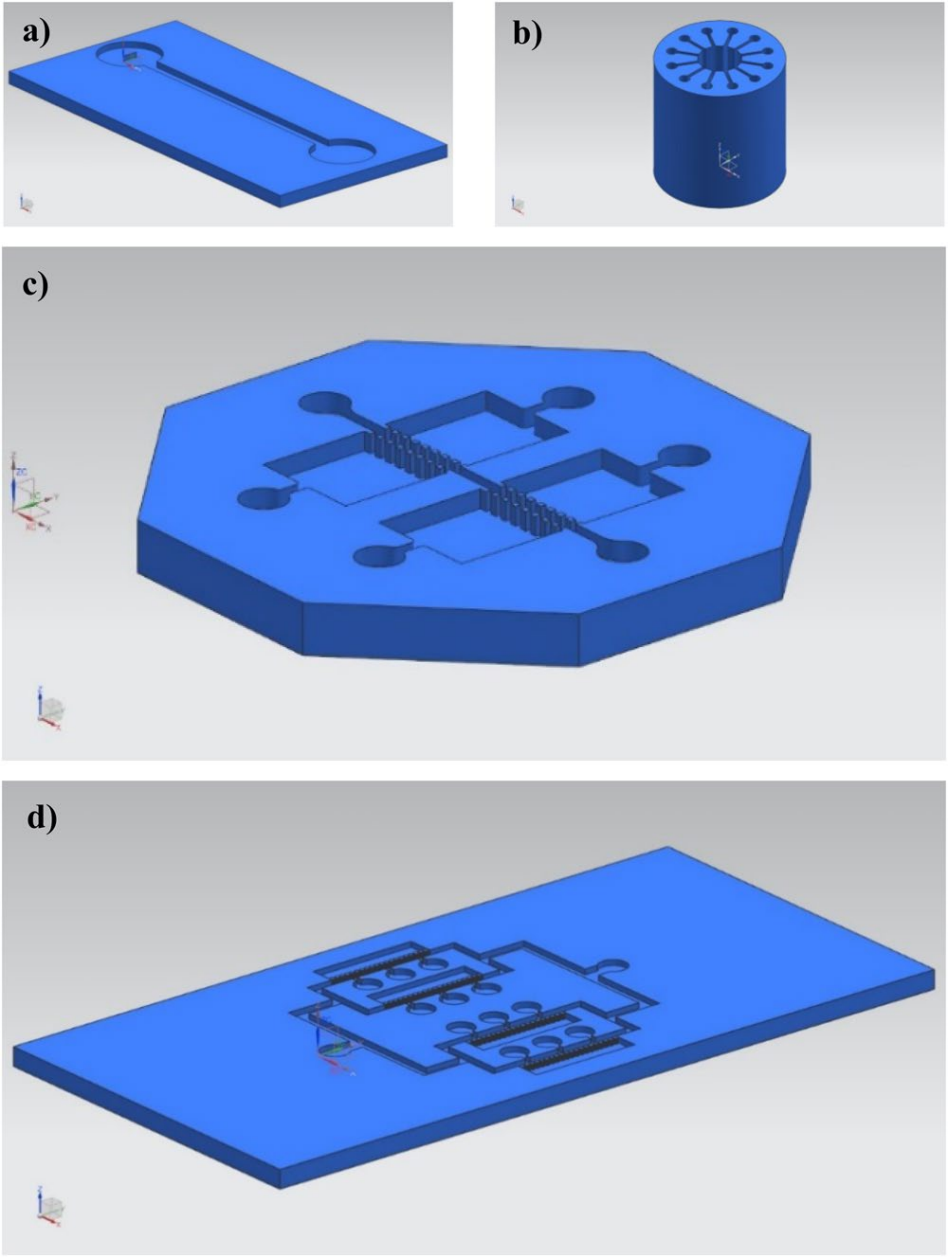
Selected set of computer-aided designs of microfluidic devices connected to lab-on-a-chip and organ-on-a-chip applications: (**a**) Simple channel connecting inlet and outlet. (**b**) Design with radial channels for modelling cell migration. (**c**) Multi-chamber microsystem with a central “vascular” channel conceived for studying metastasis “on-a-chip”. (**d**) Active layer of a biomedical microdevice aimed at modelling the blood-brain barrier.

A set of computer-aided designs was used to test the proposed simulation process. The models are shown in Fig. 3 and include: the already mentioned simple channel design (3a); a design with a central chamber and multiple radial channels, adapted from previous designs by our team and conceived for studying cell communication (3b)^26^; a multi-chamber organ-on-chip with a central vascular channel, aimed at studying metastasis (3c); and an organ-on-chip device designed for studying interactions of the blood-brain barrier also adapted from previous designs by our team (3d)^27^. In our opinion, the selected set of designs provides enough versatility to test the cellular automata in connection with real examples of labs- and organs-on-chips.

### Cell culture experiments for adjusting and validating the simulation

Two different cell lines N2A (ECACC 89121404) and MC3T3 (ECACC 99072810) were used, in the facilities of the UPM Centro de Tecnología Biomédica, which helped to adjust the growth rate of different cell types by means of Petri dish cultures and to incorporate these growth rates into the cellular automata model, running upon the multi-chamber microsystem, in which finally the cells were also cultured to validate the simulation.

Both types are adherent cells, in connection with our cellular automata model, in which the cells live, die or proliferate, but do not migrate. The N2A cells are a mouse neuroblastoma cell line with a neural and amoeboid stem cell morphology, which can differentiate into cells with features of neurons^28^, while the MC3T3 is an osteoblast precursor cell line derived from mouse calvaria^29^. Both cell types are cultured using the Dulbecco’s Modified Eagle Medium (DMEM) enriched with 10% fetal bovine serum (FBS), 2 mM glutamine 2 mM, and 1% penilicin-streptomycin and maintained in a humidified atmosphere at 37◦C and 5% CO_2_, either when initially cultured upon P24 multi-well culture plates for evaluating growth and death rates to adjust the model, or when cultured upon the microfluidic system prototype for final validation purposes. The medium was changed twice a week. Confluent cells were detached from the dishes by using Trypsin-EDTA (0.05% in HBSS, HyClone) when needed.

After counting with the support of a hemocytometer, both cell lines were seeded at 10.000 cells/cm^2^ in a P24 multi-well plate to determine the cell proliferation rates for collecting information with the cell culture plates in similar conditions to those that would be applied upon the final validation in the organ-on-chip prototype, in which the same number of cells is placed in the different inlets. Imaging and counting was also performed with the support of a Leica DMIRB inverted microscope equipped with a digital Leica DC100 camera (Leica, Nussloch, Germany). Cell viability was monitored for 11 days in P24 multi-wells and in the organ-on-chip prototype, by calcein/propidium iodide staining to analyze the number of living and dead cells and adjust the cellular automata. Cell viability was determined using a calcein/propidium iodide dual-staining assay (Invitrogen, Molecular Probes). Briefly, the cell culture medium was removed and the cells were rinsed with phosphate-buffered saline. Next, 1 μM calcein and 2 μM propidium iodide were added in each well and incubated at 37 °C for 20 minutes. Fluorescence was evaluated using an inverted Leica DMIRB microscope equipped with a digital camera, Leica DC100 (Leica, Nussloch, Germany).

## Results and Discussion

### Simulation results and potential applications of the model

**Figure 4:**
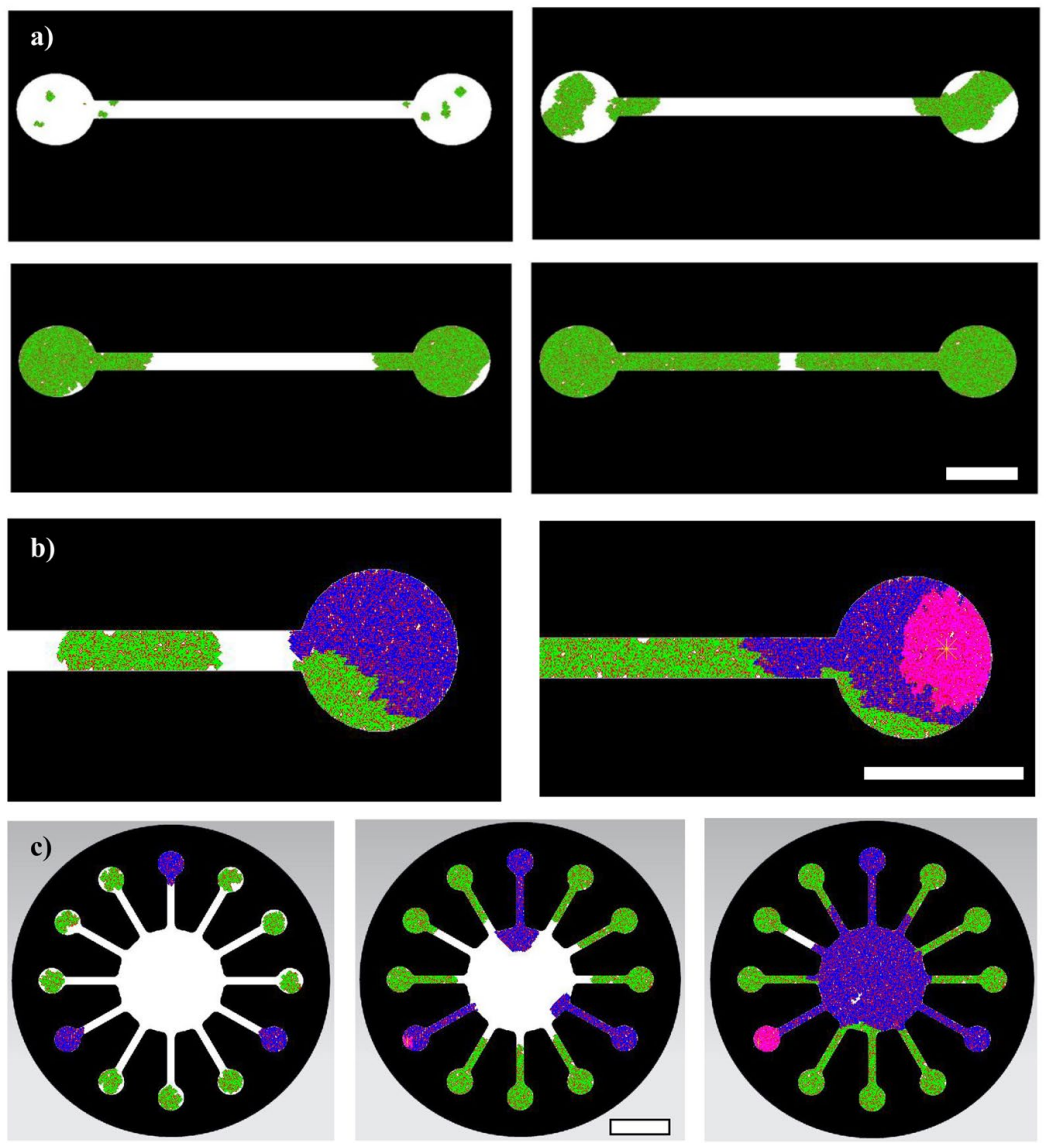
Simulation results of collective cell behaviors within different microsystems. (**a**) Cells seeded in opposite inlets and growing through a single-channel fluidic microsystems. Selected representative iterations are shown. (**b**) Detailed view of another simulation within the same single-channel device, in which two cells types -healthy (green) and invasive (blue)- are seeded and in which the addition of a drug (pink) and its diffusion, interacting just with the invasive cells, is also modeled. Selected iteration when the drug is added and aspect after addition of the drug. (**c**) Different cells seeded in radially placed inlets and proliferating along the radial channels to meet in a central chamber. Healthy (green) and invasive (blue) cells with different growth rates can be appreciated and the incorporation of a drug (pink) and its diffusion is also modeled. Selected representative iterations are shown. In all cases, the red points represent dead cells according to the defined death probabilities. For each model different representative iterations are selected to illustrate the dynamic process. Each pixel corresponds to a single cell (typically 20 × 20 μm^2^). Scale bars: 2 mm.

The procedure for defining the boundaries of cellular automata lattices directly from the CAD models is tested with different designs (see Section 2.3), upon which the collective dynamic behaviors of cells are simulated, to better expose the potential of this approach. Some results from the developed simulator performing within different devices are included in Fig. 4, which shows the results for: a) Cells seeded in opposite inlets and growing through a single-channel device; b) a detailed view of another simulation within the same single-channel device, in which two cells types -healthy (green) and invasive (blue)- are seeded and in which the addition of a drug (pink) and its diffusion, interacting just with the invasive cells, is also modeled; and c) different cells seeded in radially placed inlets and proliferating along the radial channels to meet in a central chamber. The healthy (green) and invasive (blue) cells, with different growth rates, can be appreciated and the incorporation of a drug (pink) and its diffusion is also modeled. The red points represent dead cells according to the defined death probabilities (P_d_ = 0.3 in these trials). The cellular automata is programmed so as to ask the user to select the seed zones for placing the initial cells and cell types and the stepped dynamic process can be also modified to add, at a certain step (or time), a drug or reagent, which typically interacts with one of the different cell types. In our cases we are modelling the interaction between the drug (pink) and the invasive (blue) cells. The growth rates and the diffusion speed of cells and incorporated substances can be defined and adjusted according to experimental data (see Section 3.2).

**Figure 5:**
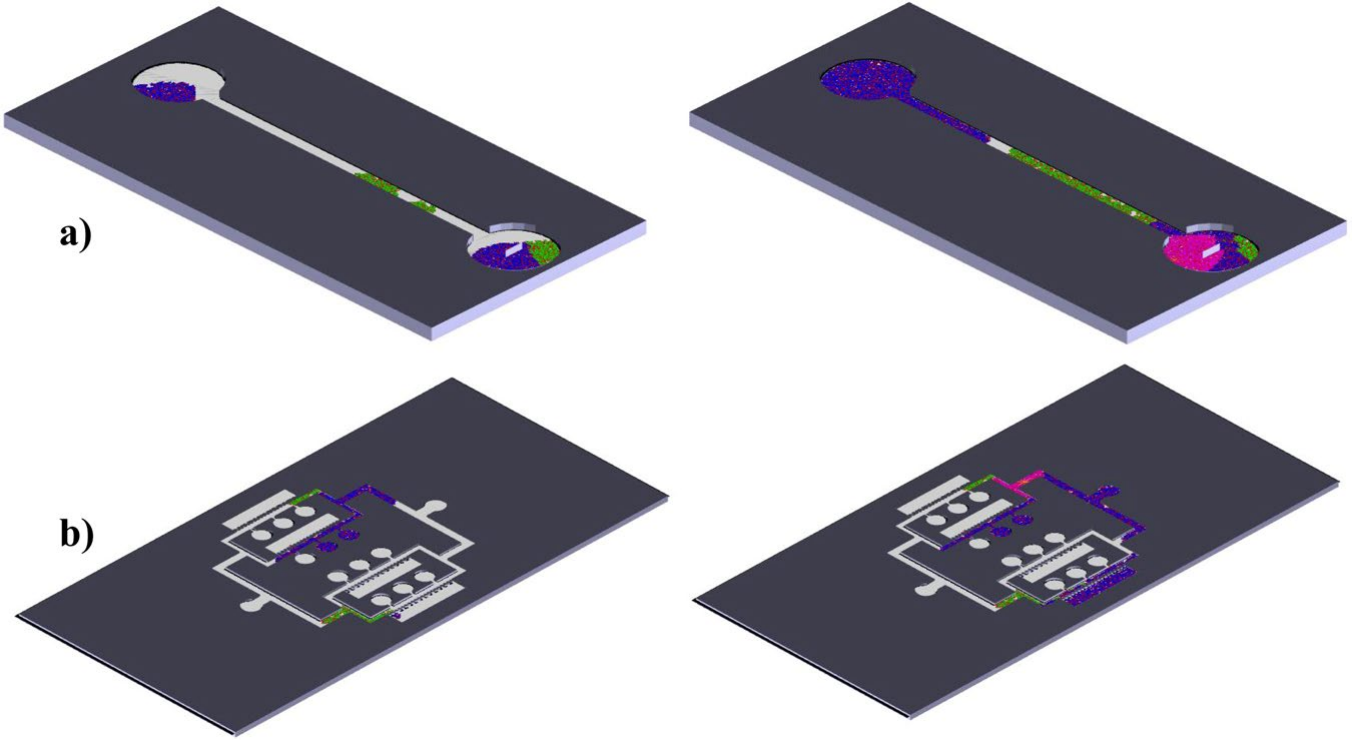
Simulation results of collective cell behaviors within different organs-on-a-chips. (**a**) 3D view of the interactions among different cell types along a single-channel microsystem. (**b**) 3D view of the interactions among different cell types within a blood-brain barrier chip. Selected representative iterations are shown. Scales: (**a**) Diameter of wells = 2 mm. (**b**) Diameter of wells = 1 mm.

**Figure 6:**
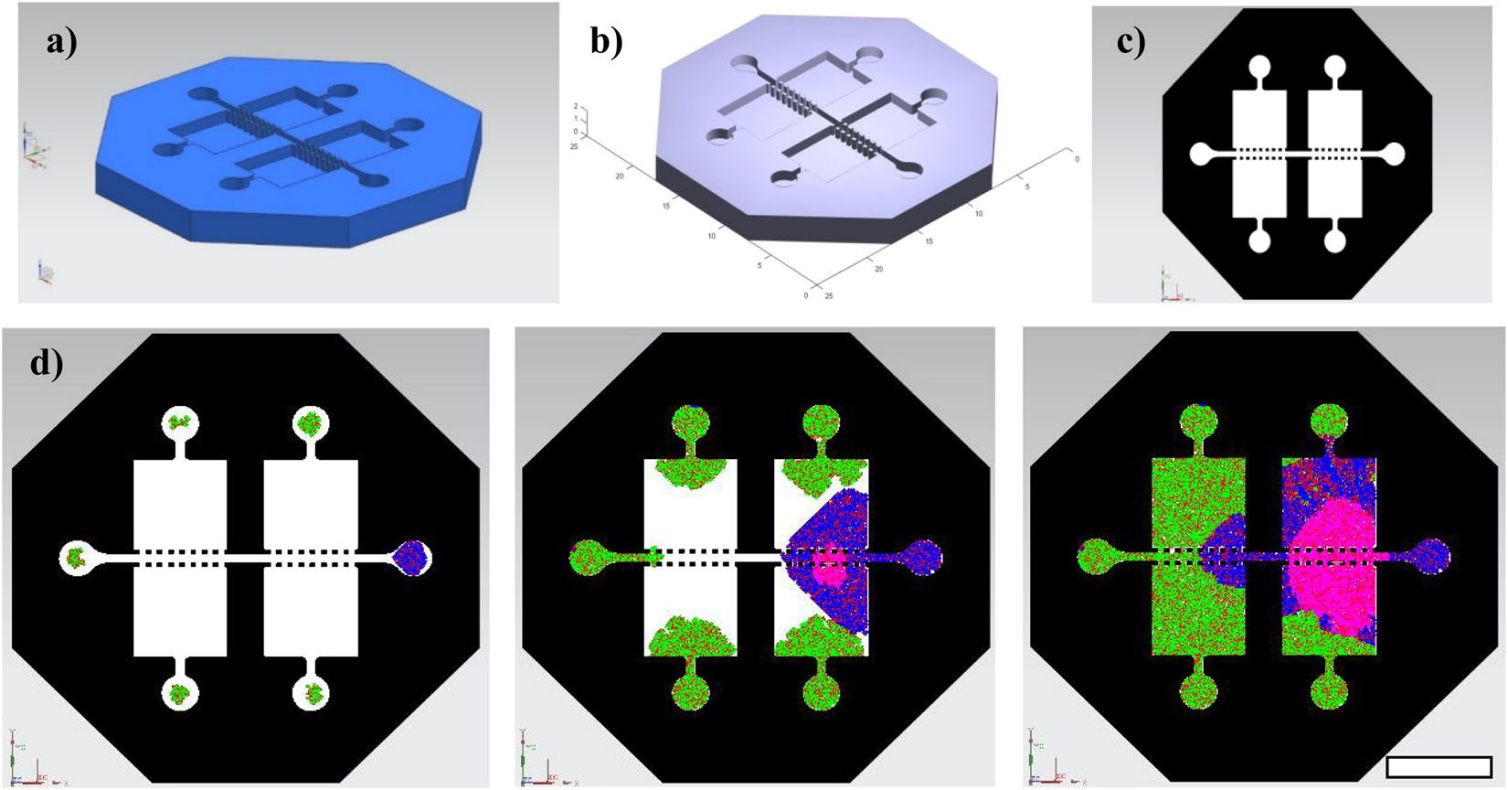
Summary of the development of a simulation process for a multi-chamber and multi-channel organ-on-a-chip device for modelling metastases: (**a**) Computer-aided design (NX-8.5). (**b**) CAD incorporated to Matlab. (**c**) Lattice with the allowed and forbidden zones. (**d**) Results from a dynamic proliferation and interaction process, which starts by seeding different cell types in the inlets (green and blue) and shows the effect of adding a drug (pink), whose diffusion and impact on cells is also modelled. Selected representative iterations are shown, including the initial state, the addition of drug and the final state after complete colonization. Scale bar: 4 mm.

A more realistic view, thanks to the use of Matlab’s CAD import tools and its three-dimensional views, is presented in Fig. 5, in which the simulation results of collective cell behaviors within different organs-on-a-chips are presented. Figure 5a shows a 3D view of the interactions among different cell types along a single channel and Fig. 5b corresponds to a 3D view of the interactions among different cell types within a blood-brain barrier chip. To illustrate the whole modelling process, Fig. 6 presents a summary of the development of a simulation for a multi-chamber and multi-channel organ-on-a-chip device conceived for studying metastases. Figure 6a shows the computer-aided design (NX-8.5) of the system and Fig. 6b shows the CAD incorporated to Matlab, while Fig. 6c presents the related lattice with the allowed and prohibited zones. The final microfluidic system, used as preliminary validation prototype, is based on the simple design of Figs 4a and 5a (mainly a microchannel connecting two inlet wells) and is obtained by PDMS casting upon a laser stereolithography mold.

### Preliminary model adjustment through cell culture experiments

**Figure 7:**
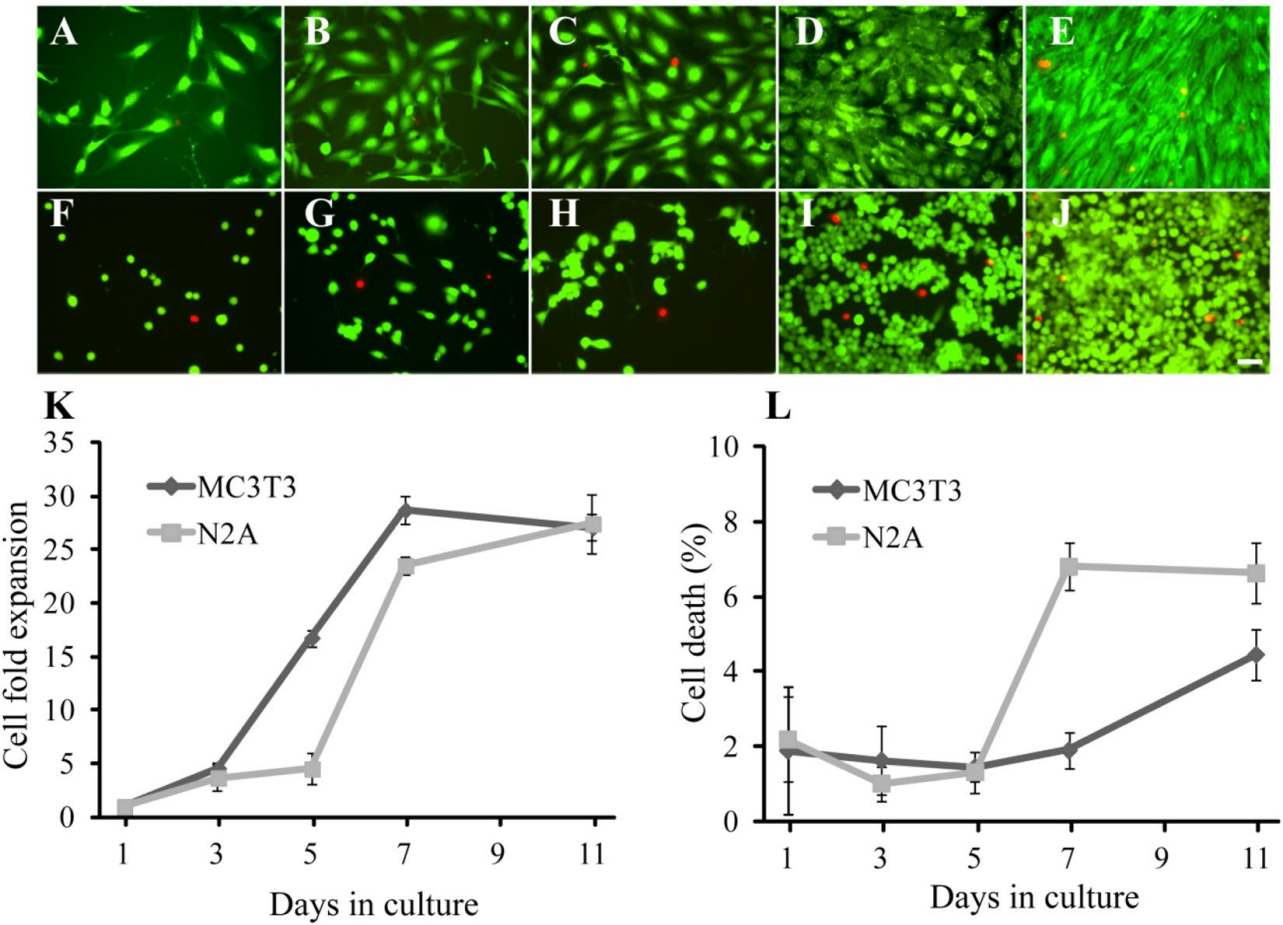
Representative microphotographs showing the evolution of MC3T3 (**A**–**E**) and N2A (**F**–**J**) cells after 1- (**A**,**F**), 3- (**B**,**G**), 5- (**C**,**H**), 7- (**D**,**I**) and 11-days (**E**,**L**) in culture. Images are shown as an example from the culture tests used to evaluate cell proliferation and cell viability dynamics. Live cells were stained with calcein (green) and dead cells with propidium iodide (red). Scale bar: 50 μm. Summary graphs show the cell growth (**K**) and cell viability (**L**) dynamics for the MC3T3 and N2A cells studied.

**Figure 8:**
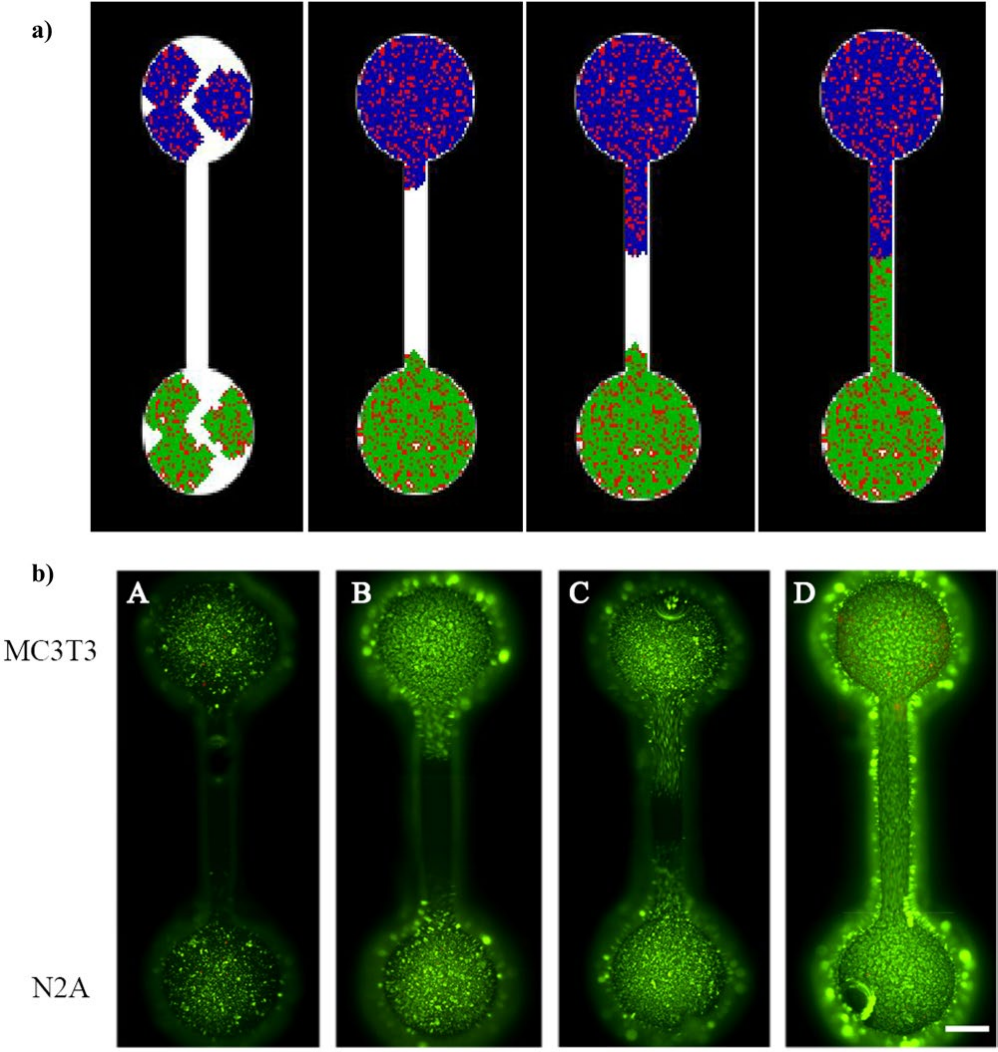
(**a**) Simulation with adjusted growth rates upon microfluidic system. Dynamic growth process along 11 days after seeding the cells through the microsystem inlets. Green: N2A (neural cells). Purple/dark blue: MC3T3 (osteoblast precursors). Red points indicate dead cells. (**b**) Actual MC3T3 and N2A cell cultures on the physical prototype of the microfluidic device. Cells were cultured and imaged at days 3(A), 5(B), 7(C) and 11(D). Live cells were stained with calcein (green) and dead cells with propidium iodide (red). Scale bar 500 μm.

Adherent cells (MC3T3 -osteoblast precursors- and N2A -neuronal phenotype-) are used to adjust the growth rates and obtain a preliminary validation, connected to actual experimental data, of the cellular automata working upon CAD-based lattices. The growth rates are calculated, as detailed below, by culturing cells in multi-well plates and monitoring cell proliferation, by taking detailed microscope images from the cultures at different times. A total of 10 fields (n = 3) for each cell type culture were photographed at days 1, 3, 5, 7 and 11. Final calcein propidium iodide staining let us analyze the number of living and dead cells and adjust the cellular automata by setting P_d_. Then, the calculated rates and probabilities are introduced in the simulator and applied to modelling the collective behavior of cells cultured within a simple microfluidic system, similar to that of Figs 4a and 5a, whose prototype is also seeded with cells to provide a experimental comparison between O-o-C simulation and O-o-C culture for a first validation of the simulator. These cell culture processes are detailed in Section 2.4 and their results further illustrated in Figs 7 and 8.

The information from the cell cultures is summarised in Fig. 7, which presents (in Fig. 7A–J) the evolution along 11 days of the MC3T3 (Fig. 7A–E) and N2A (Fig. 7F–J) cells in a representative position of the culture well, taken as example from the culture tests used to evaluate proliferation dynamics. All culture data are presented (in Fig. 7K–L) in the form of summary graph of the cell growth dynamics (Fig. 7K) and cell viability (Fig. 7L) for the MC3T3 and N2A cells studied. Mean values corresponding to different areas of the wells and their standard deviations are presented. With the information of Fig. 7K and the cell proliferation equation [eq. 1]: ${y}_{n}={y}_{n-1}+(4\times n)$, it is possible to adjust the required iterations for the different cell types in accordance with the days under culture and, hence, adjust the simulator, by employing different step numbers to trigger the proliferation step (Fig. 2a) of each cell type. The proliferation steps for each cell type, which lead to simulated cell numbers according to experimental cell culture results and are finally used for simulating the O-o-C of Fig. 6, are included in Table 1. Subsequently, Fig. 8a presents the simulation results upon the microfluidic system using the adjusted growth rates and providing a simulated overview of the dynamic growth process along 11 days after seeding the cells through the microsystem inlets. In green, the N2A cells are presented, while the MC3T3 cells are shown in dark blue; besides, the red points represent dead cells. For comparative purposes, Fig. 8b shows the actual cell culture results upon a physical prototype of the multi-chamber organ-on-a-chip device, shown in Fig. 6d, again along a 11-day cell culture process. It can be appreciated that the proliferation of the MC3T3 cells in the microsystem is faster than that of the N2A cells especially at days 5 and 7 (probably due to a lower cell adhesion of the N2A cells to the material), as also predicted in the simulation (see Fig. 8).

**Table 1:** Proliferation steps for each cell type, which lead to simulated cell numbers in accordance with the experimental cell culture results and are used to fine-tune the simulator.

	Iterations for N2A cells	Iterations for MC3T3 cells
From day 1 to 3	2	2
From day 3 to 5	3	5
From day 5 to 7	6	7
From day 7 to 9	7	8
From day 9 to 11	7	7

### Final discussion and future research proposals

Taking into account that cell culture materials and methods convey relevant cost, time and personal dedication investments, we consider that counting with simulators for predicting the collective behaviors of cells within lab- and organ-on-chip devices may prove interesting for the further expansion of the field.

We have shown that, once the cellular automata model is adjusted with the information obtained from conventional cell culture tests, the simulator helps to predict the way that cells will proliferate within the actual organ-on-a-chip system. Consequently, these simulations can prove useful for estimating the required materials, facilities and testing conditions (i.e. days under culture), so as to validate *in vitro* the real performance of innovative organ-on-chip devices and related microsystems. They can be used also for analyzing if a proposed design is adequate for studying cell-cell and cell-material interactions, in connection with disease modelling and depending on the available testing facilities. For example, the simulations from Fig. 8 helped us understand that, if we wanted to fully exploit the multi-chamber and multi-channel features of the proposed design with the cells under study, then we should extend the culture beyond the mentioned 10 days, which is challenging, or redesign with a finer or downscaled inlet, channel and chamber network. Design optimisation on the basis of the *in silico* studies is, therefore, also possible.

Among current limitations we can mention that the simulator works in a 2D environment with boundaries defined in accordance with the organ-on-chip designs and without considering cell migration, just proliferation of different species and death probabilities step-by-step. This proves enough when using adherent cells seeded at very low densities and for short culture times, but needs to be updated for including the possibility of migration and of a real 3D environment for more precise studies. We expect to solve the issue of the third dimension in a quite straightforward way, by making use of common slicing software employed for additive manufacturing by digital light processing, which generate (layer-by-layer) black and white masks, similar as the ones used in this study, but starting from complex 3D CAD files without intermediate conversion to.jpeg format^30^. Approaches from other sectors may be also employed towards three-dimensional cellular automata^31,32^ and synergies with other stochastic algorithms, such as the Monte Carlo method, may prove interesting for modelling additional phenomena within the organ-on-chip devices^33^.

Finally, we expect to enhance these simulators in the near future by using the results from simulations based on the finite-element method (FEM) as input for dynamically adjusting proliferation rates and probabilities within the lattice. In this way, fluid movement and shear rates within the microsystem and their effects on certain proliferation rates and differentiation phenomena may be modeled. The effects of thermal treatments (i.e. hyperthermia) or drug use and their impacts on cell survival may be also simulated by combining FEM and cellular automata, as we plan to study soon.

## Conclusions

Counting with simulation resources for predicting the collective behavior of cells within lab- and organ-on-a-chip devices may support the design optimisation of these innovative and useful biomedical devices and in the experimental planning for their validation. Therefore, in this study we have focused on the establishment of a direct connection between the computer-aided designs of microfluidic systems, especially labs- and organs-on-chips, and the lattices for discrete cell modelling approaches aimed at the simulation of collective cell interactions, whose boundaries can be now defined directly from the CAD models. We have illustrated the proposal using a quite straightforward cellular automata model, applied it to simulating cells with different growth rates, within a selected set of microsystem designs, and validated our approach by tuning the growth rates of different cell types with the support of cell culture experiments and by checking the results with a real organ-on-a-chip system. We call the proposed procedure “*game of life on a chip*” and we envision that similar modelling approaches may support developers of these types of medical devices to optimise their engineering-design process and to support with experimental planning for their validation and a more straightforward regulatory compliance assessment by means of selected experiments and exhaustive simulations.

## References

[1] Jenkins, G. & Mansfield, C. D. Microfluidic diagnostics: Methods and protocols. (*Springer*, New York, Heidelberg, Dordrecht, London, 2013).

[2] Waldbaur A, Rapp H, Länge K, Rapp BE (2011). Let there be chip - Towards rapid prototyping of microfluidic devices: One-step manufacturing processes. Analytical Methods.

[3] Huh D, Hamilton GA, Ingber DE (2011). From 3D cell culture to organs on chips. Trends in Cell Biology.

[4] Huh D (2013). Microfabrication of human organs-on-chips. Nature Protocols.

[5] Tsao, N. Tissue Engineering 2018–2028: Technologies, markets, forecasts opportunities for living tissue equivalents and technologies for their manufacture. *IDTechEx Research Report* (2018).

[6] Low LA, Tagle DA (2018). “You-on-a-chip” for precision medicine. Expert Review of Precision Medicine and Drug Development: Personalised medicine in drug development and clinical practice.

[7] Gizzi A (2017). Computationally informed design of a multi-axial actuated microfluidic chip device. Scientific Reports.

[8] Sove RJ, Fraser GM, Goldman D, Ellis CG (2016). Finite element model of oxygen transport for the design of geometrically complex microfluidic devices used in biological studies. PLOS One.

[9] Macklin, P. Toward computational oncology: Nonlinear simulation of centimeter-scale tumor growth in complex, heterogeneous tissues. Ph.D. Dissertation, *University of California, Irvine Department of Mathematics* (2007).

[10] Moure, A. & Gómez, H. (Advisor). Phase-field modelling and isogeometric analysis of cell crawling. Ph.D. Thesis, *University of A Coruña* (2017).

[11] Knutson, J. D. A survey of the use of cellular automata and cellular automata-like models for simulating a population of biological cells. Master Thesis. *Iowa State University Digital Repository*, 1–45 (2011).

[12] Macklin, P., Edgerton, M. E., Lowengrub, J. S. & Cristini, V. Discrete cell modelling. In Cristini, V. & Lowengrub, J. S. *Multiscale modelling of cancer: An integrated experimental and mathematical modelling approach*, Ch.6, 88–122, (Cambridge University Press, Cambridge, UK, 2010).

[13] Von Neumann, J. & Burks, A. W. Theory of self-reproducing automata” *Urbana, University of Illinois Press*, (1966).

[14] Gardner M (1970). Mathematical games: The fantastic combinations of John Conway’s new solitaire game “life”. Scientific American.

[15] Wolfram S (1984). Universality and complexity in cellular automata. Physica.

[16] Vivas J, Garzón-Alvarado D, Cerrolaza M (2015). Modelling cell adhesion and proliferation: A cellular automata based approach. Advanced Modelling and Simulation in Engineering Sciences.

[17] Lee Y, Kouvroukoglou S, Mc Intire L, Zygourakis K (1995). A cellular automaton model for the proliferation of migrating contact-inhibited cells. Biophysics.

[18] Garijo N, Manzano R, Osta R, Perez M (2012). Stochastic cellular automata model of cell migration, proliferation and differentiation: Validation with *in vitro* cultures of muscle satellite cells. Theor. Biol..

[19] Alemani D, Pappalardo F, Pennisi M, Motta S, Brusic V (2012). Combining cellular automata and lattice Boltzmann method to model multiscale avascular tumor growth coupled with nutrient diffusion and immune competition. Immunol. Methods..

[20] Graner F, Glazier J (1992). Simulation of biological cell sorting using a two-dimensional extended Potts model. Physics Review Letters.

[21] Swat M (2012). Multi-Scale Modelling of Tissues Using CompuCell3D. Computational Methods in Cell Biology.

[22] Andasari V, Roper RT, Swat MH, Chaplain MAJ (2012). Integrating intracellular dynamics using CompuCell3D and Bionetsolver: Applications to multiscale modelling of cancer cell growth and invasion. PLOS One.

[23] Summers, R., Abdulla, T. & Schleich, J.-M. Advances in modelling of epithelial to mesenchymal transition. *XIII Mediterranean Conference on Medical and Biological Engineering and Computing* 1225–1228 (2013).

[24] Rosa L, Pareja D, Perez F, Domech D, Mendez A (2015). Experiences in the use of CompuCell3D in the career of Biomedical Engineering. IFMBE Proceedings.

[25] Díaz Lantada, A. *et al*. Rapid prototyping of biomedical microsystems for interacting at a celular level. Chapter 8, 125–156, In *Diaz Lantada, A. Handbook on Microsystems for Enhanced Control of Cell Behavior: Fundamentals, Design and Manufacturing Strategies, Applications and Challenges*. Springer, ISBN 978-3-319-29328-8 (2016).

[26] Díaz Lantada, A., Bustamante, A., Morss Clyne, A., Urbano, R. & Canver, A. C. Overview of microsystems for studying cell behavior under culture. Chapter 12, 201–220, In *Diaz Lantada, A. Handbook on Microsystems for Enhanced Control of Cell Behavior: Fundamentals, Design and Manufacturing Strategies, Applications and Challenges*. Springer, ISBN 978-3-319-29328-8 (2016).

[27] Díaz Lantada, A. *et al*. Overview of microsystems for studying cell behavior under culture. Chapter 22, 201–220, In Diaz Lantada, A. *Handbook on Microsystems for Enhanced Control of Cell Behavior: Fundamentals, Design and Manufacturing Strategies, Applications and Challenges*. Springer, ISBN 978-3-319-29328-8 (2016).

[28] Salto R (2015). β-Hydroxy-β-Methylbutyrate (HMB) promotes neurite outgrowth in Neuro2a Cells. PLoS ONE.

[29] Kodama HA, Amagai Y, Sudo H, Kasai S, Yamamoto S (1981). Establishment of a clonal osteogenic cell line from newborn mouse calvaria. Japanese Journal of Oral Biology.

[30] Example of slicer for digital light processing: https://github.com/formlabs/hackathon-slicer.

[31] Zygourakis K, Markenscoff PA (1996). Computer-aided design of bioerodible devices with optimal release characteristics: A cellular automata approach. Biomaterials.

[32] Mardiris V, Sirakoulis GC, Mizas C, Karafyllidis IA (2008). CAD system for modelling and simulation of computer networks using cellular automata. IEEE Transactions on Systems, Man, and Cybernetics, Part C (Applications and Reviews).

[33] Erkizia G, Rainer A, De Juan-Pardo EM, Aldazabal J (2010). Computer simulation of scaffold degradation. Journal of Physics: Conference Series, Surface Modifications and Functionalisation of Materials for Biomedical Applications.

